# Switching from weakly to strongly limited injection in self-aligned, nano-patterned organic transistors

**DOI:** 10.1038/srep31387

**Published:** 2016-09-27

**Authors:** Karin Zojer, Thomas Rothländer, Johanna Kraxner, Roland Schmied, Ursula Palfinger, Harald Plank, Werner Grogger, Anja Haase, Herbert Gold, Barbara Stadlober

**Affiliations:** 1 Institute of Solid State Physics, Graz University of Technology and NAWI Graz, Petersgasse 16, 8010 Graz, Austria; 2 MATERIALS – Institute for Surface Technologies and Photonics, Joanneum Research Forschungsgesellschaft mbH, Franz-Pichler-Strasse 30, 8160 Weiz, Austria; 3 Graz Centre for Electron Microscopy and Institute for Electron Microscopy and Nanoanalysis, Graz University of Technology, Steyrergasse 17, 8010 Graz, Austria

## Abstract

Organic thin-film transistors for high frequency applications require large transconductances in combination with minimal parasitic capacitances. Techniques aiming at eliminating parasitic capacitances are prone to produce a mismatch between electrodes, in particular gaps between the gate and the interlayer electrodes. While such mismatches are typically undesirable, we demonstrate that, in fact, device structures with a small single-sided interlayer electrode gap directly probe the detrimental contact resistance arising from the presence of an injection barrier. By employing a self-alignment nanoimprint lithography technique, asymmetric coplanar organic transistors with an intentional gap of varying size (< 0.2 μm) between gate and one interlayer electrode are fabricated. An electrode overlap exceeding 1 μm with the other interlayer has been kept. Gaps, be them source or drain-sided, do not preclude transistor operation. The operation of the device with a source-gate gap reveals a current reduction up to two orders of magnitude compared to a source-sided overlap. Drift-diffusion based simulations reveal that this marked reduction is a consequence of a weakened gate-induced field at the contact which strongly inhibits injection.

The quest for flexible, light-weight, unbreakable electronics at relatively low cost triggered intense research on organic thin-film transistors (OTFTs) that are an integral part of applications like integrated circuits^1^,^2^, RFID tags^3^,^4^ and OTFT-driven display backplanes^5^,^6^. The steep rise in switching speeds or on-current level is due to the development of better performing materials and careful consideration of critical device geometry parameters^7^. In particular for boosting the speed of operation it is crucial to improve not only the transconductance, but to simultaneously minimize the parasitic capacitance due to interlayer electrode overlaps, i.e., geometric overlaps between source/drain and gate electrode^8^,^9^,^10^. Considering that self-alignment patterning techniques^11^,^12^,^13^,^14^,^15^ approach the limit of nearly zero overlap^9^,^10^ the questions arises, how OTFTs operate if structuring techniques cause the formation of a small gap between the gate and source electrode rather than an overlap. This apparently technology-driven question attains, in fact, fundamental importance when considering a crucial aspect in which OTFTs differ from their inorganic counterparts: The operation of thin-film transistors relies on controlling the current via accumulation of charges at the semiconducting-insulator interface due to a gate-source bias. However, at least as important is the ability to efficiently inject charge carriers, since pristine organic semiconductors lack mobile charges due to their wide band gap^7^,^8^. If an injection barrier is present, the gate-source bias controls also the injection current, since local, essentially gate-induced fields near the contact lower the Schottky barrier^16^,^17^,^18^. Opening a gap between source and gate electrode can thus be expected to affect both fundamental requirements, because the electric field available, (i), for charge accumulation and, (ii), for lowering the Schottky barrier is inevitably reduced. The impact of the gap is likely to be particularly severe in coplanar transistor architectures. As source and drain electrodes are located directly at the interface between the organic semiconductor (OSC) and the dielectric layer (as depicted in Fig. 1a), charge injection and transport occur, in essence, exclusively along this interface. Thus, a small gap weakens the gate-induced electric field in a confined but most decisive region, i.e., where the two field-dependent processes of injection and accumulation ought to take place.

In this work, we utilize such an asymmetry in the interlayer electrode overlap of co-planar transistor structures (depicted in Fig. 1a) to establish a direct comparison of the device current with and without a gap between the gate and the injecting electrode.

Thus, we expect to directly obtain the fingerprint and extent of losses in driving voltage that are caused by hindrance of injection across Schottky barriers. Such losses are known to markedly contribute to the highly undesirable contact resistance^16^,^17^,^18^,^19^. However, the quantification of this effect on the transistor characteristics is challenging due to the presence other superimposed contact-related losses, in particular for short-channel, high performance transistors^20^. Operating the asymmetric devices now with either the overlap-sided contact or the gap-sided contact as injecting source, the operation will (i) either fully benefit from the gate-induced field or (ii) is expected to be suppressed due to a field being impaired in a very confined region close to the injecting contact. Extrinsic factors possibly affecting the charge transport, be that local morphologies, trap distributions, grain boundaries, local fluctuations due to, e.g., interactions between the semiconductor and the dielectric, access contact resistances etc., remain exactly the same irrespective the “direction” of operation.

## Results

### Modified self-alignment nanoimprint lithography

**Figure 1:**
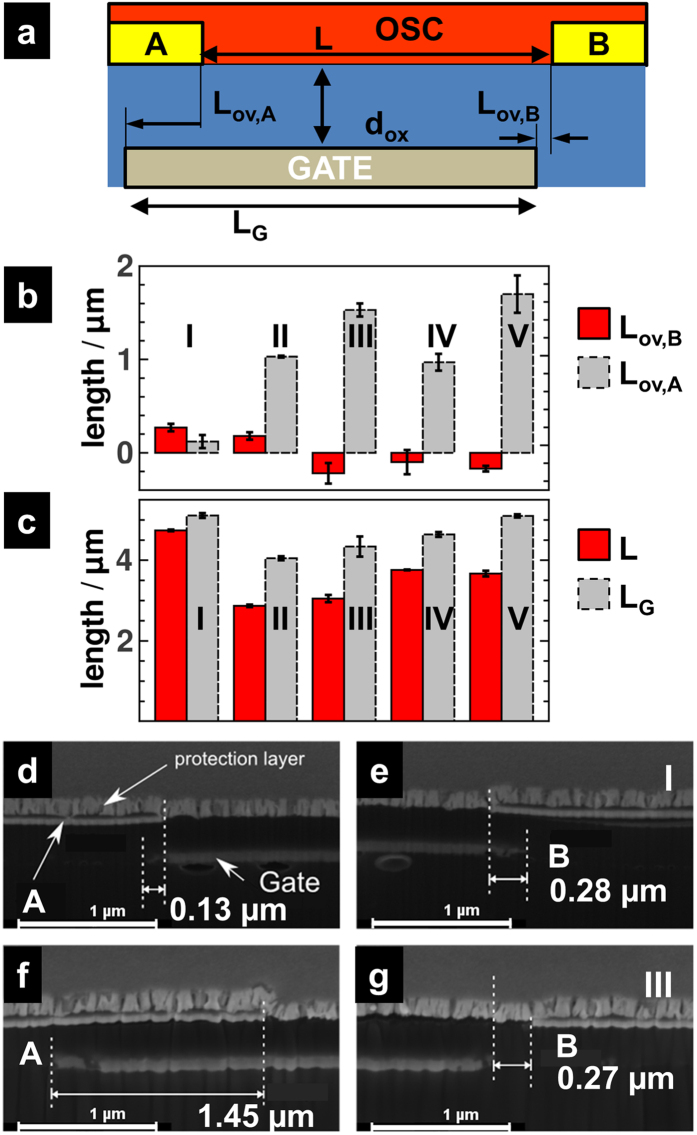
(**a**) Schematic cross-section of a coplanar OTFT with a gate partially overlapping with the source and drain contacts; both contacts A or B can be employed as injecting source contact; (**b**,**c**) Overlap between electrode and gate on side A, *L*_ov,A_, and side B, *L*_ov,B_, (**b**), and channel length, L, and gate extension, *L*_G_ (**c**), obtained for five different contact alignments **I**-**V**. (**d**–**g**) SEM images of a cross section of device **I** (**d**,**e**) and **III** (**f**,**g**); (**d**,**f**) show the measurement of side A overlap, (**f**,**g**) of side B overlap.

The devices were fabricated utilizing nanoimprint lithography (NIL) as a high-resolution top-down patterning technique. The method is exploited to fabricate devices for electrical, optical, photonic and biological applications. A topographic micro- or nanoscale pattern is transferred from a stamp into a UV-curable resist by pressing the stamp into the liquid resist layer. Simultaneous UV curing fixes the pattern. This pattern is used either as an etch mask or as a sacrificial layer for a subsequent lift-off processes. With NIL structures with resolutions down to 10 nm, aspect ratios up to 20 and high yields were realized, the latter being mainly determined by the defect density of the stamp^21^,^22^. An associated, yet very intriguing aspect of NIL is that it can be done in a parallel mode; the latter is a necessary precondition to translate NIL into a high-throughput roll-to-roll process. This parallel processing has been demonstrated quite recently by our group with high quality and yield^23^ and promotes NIL to an ideal patterning technique for large-area organic and printed electronics. For the purpose of asymmetric transistors, we modified the self-alignment nanoimprint lithography (SANIL) process^9^ such that the interlayer electrode overlaps lengths (i.e., on side A and B) of coplanar OTFTs can be varied systematically and changed from a gap to an overlap situation. In essence, the gate electrode acts as a photo mask during the photolithography to define the source/drain electrodes. This SANIL process was decisively altered in one step: In order to control and modify the source/drain to gate overlap, the angle of the sample with respect to the UV light is varied by placing the samples on wedges with varying inclinations. Gold contacts were evaporated on a PVCi (polyvinylcinnamate) layer in the predefined source and drain positions and, finally, a pentacene layer was evaporated on top. (cf. methodology section for further details). For one representative device associated to each of the five implemented inclinations, a cross section was prepared by focused-ion-beam (FIB) milling. The geometry parameters deduced from TEM images of the cross sections for all five inclinations are summarized in Fig. 1b,c. Alongside Fig. 1d–f shows representative TEM images for the devices **I** and **III**. As can be seen from Fig. 1b, the overlap *L*_OV,A_ increases on side A from 180 nm for device **I** to 1.7 μm for **V**. At the same time, the overlap on side B, *L*_OV,B,_ turns from a 270 nm overlap (**I**) to a 220 nm gap (**V**). Note that we refer to electrode gaps by using negative overlap values *L*_OV_. Thus, there are devices with non-equal overlaps on each side (**I** and **II**) and devices possessing an electrode overlap exceeding 1 μm and, on the opposing side, a gap ranging from 100 to 220 nm (**III**-**V**). Note that the gate lengths are with 4 and 5 μm dependent on the inclination chosen during UV structuring. This dependence is attributed to variations in the etch rate of the Cu gate. The even larger variation in channel length between 2.8 and 4.7 μm (see Fig. 1c) is attributed to non-ideal compensation of the exposure dose for inclined incidence angles.

### Transistor characteristics

Each device is electrically characterized in two configurations: In a first measurement, electrode A is used as source and electrode B as drain electrode (cf. Fig. 1a). In a second measurement, the roles of the electrodes were exchanged.

**Figure 2:**
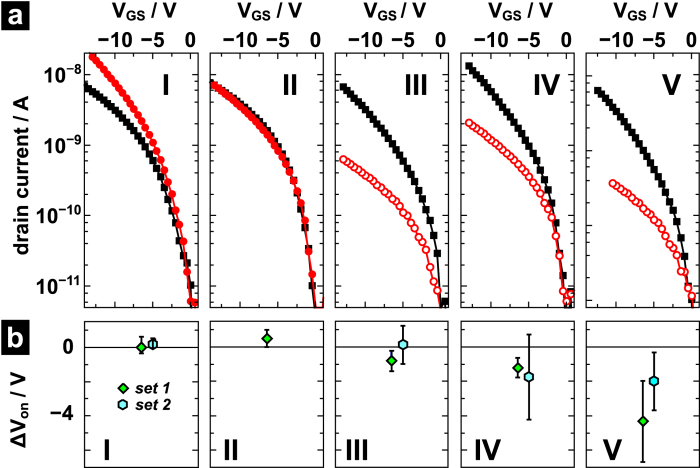
(**a**) Transfer characteristics for alignment types indicated Fig. 1b,c corrected for the onset voltage V_on_. Shown is the drain current when operating the device with the source on side B (circles), i.e., with a gap to the gate electrode (open circles) in devices **III**-**V**, and with the source on side A (squares). Note that devices **II** and **III** as well as **IV** and **V** have comparable channel lengths. V_DS_ = −14 V for all measured curves; (**b**) Difference between onset voltages, ΔV_on_ = V_on,B_ − V_on,A_, and its standard deviation for two batches of devices (diamonds and hexagons). The channel width of the devices is W = 150 μm.

Figure 2a compiles the transfer characteristics *I*_DS_(V_GS_) obtained for the two measurement configurations in the saturation regime for all the alignment types **I**-**V**. To directly compare the drain-source currents from the two configurations, all curves have been onset voltage corrected (cf. Supplementary Figure S2 for uncorrected curves).

Devices **I**-**V** operated with a source-gate overlap possess on-currents in saturation of about 3×10^−8^ A at V_DS_ = −14 V (black squares in Fig. 2) with mobilities of about 10^−3^ cm^2^/Vs. To justify that these overlap configurations are a reliable reference to assess the impact of the contact gap, we need to ensure that, (i), the devices do not suffer from hysteresis effects and, (ii), the impact of the overlap length is assessed. Therefore we turn to devices **I** and **II**, i.e., the devices possessing a source-gate overlap in each configuration (Fig. 1a,c). In case of device **II**, the steady-state currents associated to the two configurations coincide despite very different overlap lengths *L*_OV,A_, *L*_OV,B_ and indicate a vanishing hysteresis (Fig. 2). Device **I** has by far the smallest total overlap of the gate with the electrodes A and B (Fig. 1b). A closer inspection of the FIB results obtained in particular for the small overlap *L*_OV,A_ = 120 nm reveals that the uncertainty associated to the determination of *L*_OV,A_ is with ca. 140 nm as large as the value of *L*_OV,A_ itself (error bars in Fig. 1b). Considering that this uncertainty has been determined from evaluating different FIB cross-sections of the device, we cannot rule out that certain locations within the device possess, in fact, a gate-source gap rather than an overlap. This might explain why the drain current in **I** obtained for a source contact on side A is slightly lower than for the opposite configuration (Fig. 2).

We, therefore, rather focus on device **II** as an overlap-only reference, thanks to which we can confidently assign changes in the configuration-dependent drain currents to the presence or absence of a gate-source electrode overlap. Devices **III-V** exhibit transistor-like behavior when operated with a gap between the source (contact B in Fig. 1a) and gate electrode, even though the currents drop up to one order of magnitude compared to the other operation configuration (circles in Fig. 2a). These reductions are consistent with the observations made in Si-MOSFETs^24^ and double gate OTFTs^25^ with mismatched gate and source electrodes. Device **III** is suited best for quantitative comparison to the reference device **II**, since it possesses a comparable channel length L and also a gate-source overlap exceeding 1 μm. The current obtained for overlap operation (source being contact A in Fig. 1a) is nearly the same for both devices (squares in Fig. 2). In gap-configuration with a gap of *L*_OV,B_ = −220 nm, **III** yields a current being a factor of 10 smaller than in the overlap configuration (*L*_OV,B_ = 180 nm) of **II** (circles in Fig. 2a). Increasing the gate-source gap, e.g., by going from 100 nm in **IV** to 170 nm in **V** (**IV** and **V** are comparable due to similar gate lengths), further reduces the current with respect to the gate-source overlap operation; the reduction increases by a factor two from **IV** to **V** (Fig. 2a).

For each device, we also determined the difference in onset voltage ∆V_on_ = V_on,B_ − V_on,A_ between the two configurations. The average of ∆V_on_ and the standard deviation of each alignment type **I**-**V** is depicted in Fig. 2b (diamonds). Both overlap-only device types **I** and **II** exhibit a small positive shift in V_on_ of less than 0.7 V. Devices **III**-**V**, on the other hand, exhibit a shift towards more negative V_on_ values (exceeding |∆V_on_| of reference device **II**), when going from overlap to gap operation. Moreover, comparing devices **IV** and **V** of similar channel lengths suggests that the shift is more pronounced for the larger source-gate gap in **V**. Since the data spread within the originally fabricated set of devices is rather large (set 1, diamonds in Fig. 2b), the whole experiment was carefully repeated and a total of 13 OTFTs associated to **I**, **III**-**V** were electrically characterized. All current voltage characteristics presented in Fig. 2a and the trends in ∆V_on_ were reproduced in the second set of devices (set 2, hexagons in Fig. 2b).

### Simulation of the transfer characteristics

To rationalize the device operation in the gap configuration and to explain the trends with respect to the gap extension, we utilized two-dimensional drift-diffusion based simulations. Particular care has been taken to properly account for the field-dependent charge injection at the contacts. The details of the simulation approach are given in the Methodology section. We pick device **IV** as representative example and take its geometric values as the input for the simulation. Here the gap between source and gate amounts to 100 nm, i.e., less than three percent of the total channel length of 4 μm. The dielectric layer being 337 nm thick is followed by a 100 nm thick gate electrode; both values were determined from the FIB cross-sections. To demonstrate the impact of the mobility on the device current, we assumed a constant mobility of either μ = 10^−3^ cm^2^V^−1^s^−1^ corresponding to the experimentally obtained values for devices **I**-**V** or μ = 1 cm^2^V^−1^s^−1^. For a more convenient comparison of the transfer curves obtained for the different mobilities μ, in particular with the expectations from the Gradual Channel Approximation (GCA), we turn to scaled drain currents *i*_DS_ = *I*_DS_μ^−1^*W*^−1^ (*W* being the transistor width). For ideal devices, i.e., devices in which the GCA holds due to the absence of a source-gate gap and an injection barrier, this scaled current *i*_DS_ is the same independent of the mobility value and, of course, of the measuring configuration. Thus, the corresponding transfer curves *i*_DS_-V_GS_ will lie exactly on top of each other. The reduced current in gap operation may either result (i) from a weakened accumulation within the gap or (ii) from a reduced injection of holes across the Schottky barrier due to a profoundly weakened electric field near the source contact.

**Figure 3:**
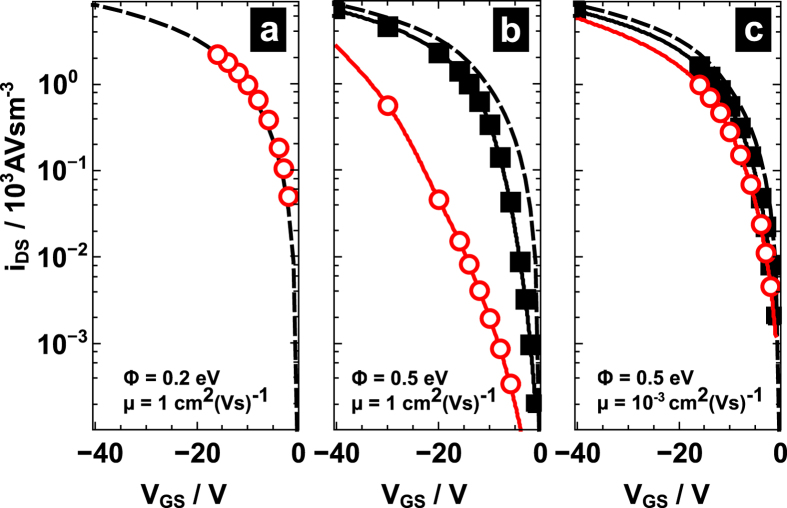
Simulated drain-source current, i_SD_, scaled with the inverse mobility as a function of the gate bias in device structure **IV** operated either with a source-gate gap of *L*_ov,B_ = −100 nm (open circles) and a source-gate overlap of *L*_ov,A_ = 1 μm (closed squares) for, (**a**), an injection barrier of 0.2 eV and μ = 1 cm^2^V^−1^s^−1^ for gap operation; (**b**) as in (**a**) for gap and overlap operation with higher injection barrier 0.5 eV corresponding to Au-pentacene; (**c**), as in (**b**) with a lower mobility μ = 10^−3^ cm^2^V^−1^s^−1^. For comparison, the corresponding value derived from the gradual channel approximation is shown (dashed line). The devices were operated at V_DS_ = −14 V.

To explore the first scenario, we performed a simulation assuming perfect injection, i.e., the injection barrier height Φ, defined as the offset between the Fermi level in the metal and the transport level in the OSC, is set to a small value of 0.2 eV. Moreover, a large mobility of 1 cm^2^V^−1^s^−1^ is assumed. Due to the large current demand associated to such large mobilities, the consequences of imperfections near the contacts are expected to be larger pronounced than for small mobilities. It is shown in Fig. 3a that the resulting transfer curve (at V_DS_ = −14 V) for gap operation (circles) essentially coincides with the curve expected from GCA (dashed line). Despite a small gap segment, charges spread and accumulate across the entire channel as in the ideal case.

The second possible scenario relates to the presence of an appreciable injection barrier. Figure 3b depicts the currents for gap (source = contact B) and overlap (source = contact A) operation assuming an injection barrier of 0.5 eV to represent the Au pentacene interface^26^,^27^,^28^ and a high charge mobility of 1 cm^2^V^−1^s^−1^. It is readily seen that the current obtained with an overlap to the gate (squares) is consistently larger than in the gap case (circles). The largest difference between the gap and overlap currents of almost two orders of magnitude is established near V_DS_ = V_GS_, i.e., near V_GS_ = −14 V. A closer inspection reveals that the transfer curves differ mainly in threshold voltage and, to a much smaller extent, in the slope (*I*_DS_(V_GS_) plots on a linear scale are shown in the Supplementary Figure S3). Moreover, it is remarkable, that the overlap curve (squares) is almost recovering the current predicted from GCA (dashed line). Note here that our simulations indicate that the drain-source current with drain gap is even superior to the current obtained with a gate fully overlapping drain and source contacts. This current enhancement is ascribed to an improved current collection and, correspondingly, altered current spreading^29^ at the drain due to a lessened influence of the gate-induced field directly at the drain electrode.

When reducing the mobility by going from 1 to 10^−3^ cm^2^V^−1^s^−1^, the discrepancy between gap and overlap operation reduces to a factor of ca. 2 at V_GS_ = −14 V (Fig. 3c). Clearly, the device operation is affected by the gap when an injection barrier is present. However, the extent of the effect is also dependent on the subsequent charge transport in the channel.

## Origin of hindered injection in the gap configuration

**Figure 4:**
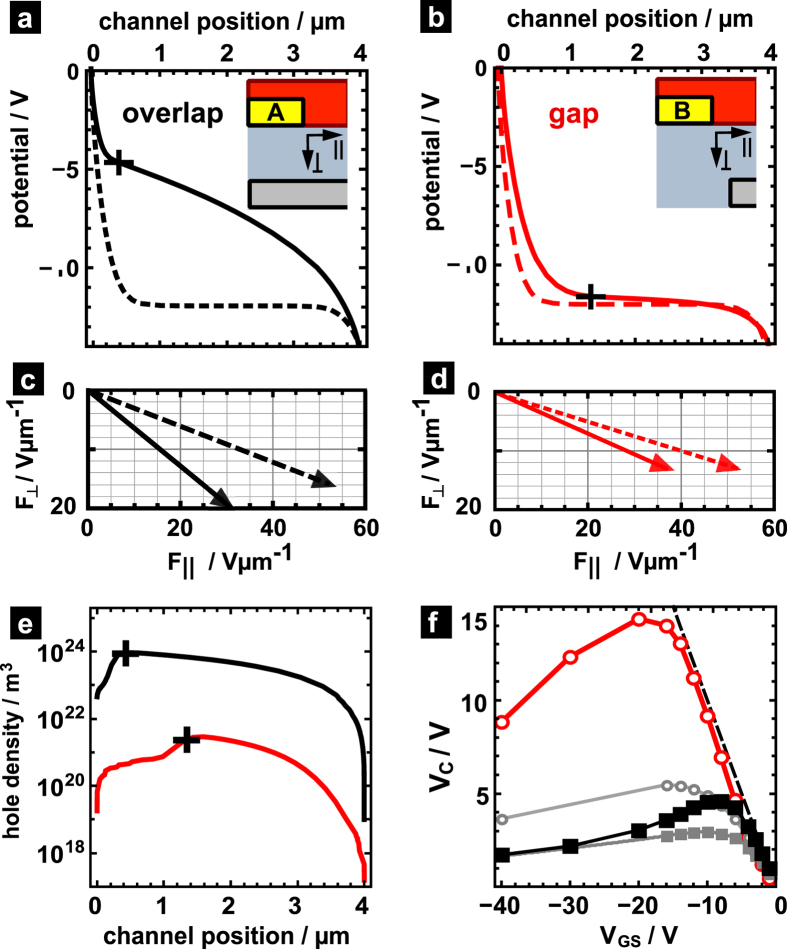
(**a**,**b**) Electrostatic potential at the semiconductor-dielectric interface, namely the steady-state potential (solid line) and the potential prior starting the injection of charges (dashed line) for (**a**) an overlap and (**b**) a gap between source and gate for the operation at V_DS_ = −14 V and V_GS_ = −12 V with an injection barrier of 0.5 eV and a mobility of 1 cm^2^V^−1^s^−1^. (**c**,**d**) Average electric field in the steady state (solid arrow) and initial state (dashed line) present in the region extending 100 nm from the position of the source electrode towards the drain. (**e**) Steady-state hole distribution at the interface for the overlap (black) and gap situation (red). The crosses mark the channel position at which depletion turns into accumulation. (**f**) Contact voltage V_C_ as a function of the gate bias for a gap (open circles) and an overlap (filled squares) between source and gate for a mobility of μ = 1 cm^2^V^−1^s^−1^ (black symbols) and 10^−3^ cm^2^V^−1^s^−1^ (grey symbols). As a guide to the eye, the relation V_C_ = |V_GS_| is indicated by a dashed line.

To explain the interplay between injection at the contact and charge transport, we first inspect the local potential and hole density distribution for a situation in which the currents differ markedly. Figure 4 compares the potential distribution (solid lines in Fig. 4a,b) and the hole distribution (Fig. 4e) along the dielectric-semiconductor interface at V_GS_ = −12 V. In all plots, the injecting contact, i.e., the source contact, is placed on the left side; the edge of the source contact corresponds to channel position zero.

In the reference case of operation with overlap, shown as black line in Fig. 4a, the potential exhibits a pronounced drop prior turning into a prototypical channel-like behavior, i.e., a typical quadratic spatial dependence within the channel^30^. The potential drop near the source contact defines a contact voltage V_C_. The value of V_C_, here ca. 4.2 V, is taken as the difference between the true potential and the channel potential that is extrapolated beyond the channel at the position of the source contact^18^. The position up to which the true potential differs from the extrapolated channel behavior is marked by a cross in Fig. 4a (ca. 500 nm). The contact potential is associated to a local field that lowers the Schottky barrier at the contact and, thus, permits an injection current being larger than the nominal barrier height would let us expect. The amount of V_C_ is established such that the current being injected in the presence of this contact voltage matches the space-charge-limited current driven by the remaining voltage in the channel, i.e., the drain-source bias in excess of V_C_^17^.

Associated to the occurrence of V_C_ is the formation of a depletion region in which the carrier density, indicated with a black line in Fig. 4e, is at least an order smaller than in the channel starting at ca. 500 nm (cf. position of the cross in Fig. 4e). Note that the rather large extension of this depletion zone is a consequence of a small gate-induced field at the interface due to a rather small capacitance per unit area C′ = 7 nF of the dielectric layer^31^.

When going to the case of a gap between source and gate, shown as solid line in Fig. 4b, we recognize a strong increase in V_C_ and a widening of the depletion zone to 1.4 μm. In this point of operation, we encounter the extreme case of V_C_ approaching the value of |V_GS_|. Thus, the gate bias in excess of V_C_, i.e., the effective gate bias left to accumulate charges in the channel region, is strongly reduced so that the density of holes in the channel is three orders of magnitude smaller than in the overlap case (red line in Fig. 4e).

Recalling that introducing a gap between source and gate is expected to reduce the local electric field at the contact facet, it appears to be counterintuitive that we, in fact, encounter larger electric fields due to an increased V_C_. To resolve this finding, it is best to consider the evolution of the potential from the initial state to steady state. In the initial state, the external biases are applied but no mobile charges are present in the device yet. Now the presence or lack of an interlayer electrode overlap ought to be clearly detectable. The corresponding potentials along the channel are shown in Fig. 4 (dashed lines) for the overlap (Fig. 4a) and the gap (Fig. 4b) situation, respectively; in each case, the initial potential drop between the source and the channel amounts to V_C_ = |V_GS_|. The initial average electric field present in a 100 nm wide region next to the source contact, indicated as dashed arrow in Fig. 4c,d, possesses in gap configuration a clearly smaller component *F*_⊥_ perpendicular to the semiconductor-dielectric interface (compare the *F*_⊥_-component of the solid arrows in Fig. 4c,d). However, the initial parallel field component *F*_||_ being responsible for reshaping the injection barrier, is hardly affected by the interlayer electrode overlap (compare the *F*_||_ -component of the dashed arrows in Fig. 4c,d). In the course of operation, charges enter the device across the barrier, accumulate at the organic-dielectric interface and, thus, partially screen the initial potential drop. The more charges are injected, the larger the screening and the smaller the steady-state potential drop V_C_ eventually becomes. This is well illustrated in Fig. 4c, in which the average steady-state field next to the source (solid arrow) is significantly altered due to the accumulated charges; *F*_||_ reduces due to screening and *F*_⊥_ increases due to the buildup of an interface charge density. In turn, that implies for situations, in which even the initial field is not able to sufficiently lower the injection barrier, a marked reduction of the initial potential drop V_GS_ is precluded due to inefficient accumulation. Such a lack of accumulation is encountered in the gap configuration. As seen in Fig. 4d, injected charge carriers reduce the field *F*_||_ to some extent (cf. solid and dashed arrow), but accumulate poorly at the interface due to the weak field *F*_⊥_. Widening the gap further leads us, therefore, to expect that a weakening of the accumulation field across a larger distance from the source will further reduce the accumulated charge density and, thus, the steady state current. Our line of argumentation is further corroborated by a recent experimental finding. By utilizing photocurrent microscopy, the electric field strength near the injecting source contact was probed for the two possible directions of operation of an asymmetric OTFT^32^. When operating in the direction yielding the inferior drain current, the electric field strength is demonstrated to be much higher than for the direction giving superior current.

Obviously, the gap configuration does not reside in the mode of insufficient injection when going to larger gate biases. Then, the drain-source currents increase appreciably and the device is clearly in an on state. To study how the device operation alters when going from low biases to the linear regime, we compare the evolution of the contact voltage V_C_ as a function of the gate bias V_GS_ for both the gap and overlap configuration in Fig. 4f. The values of V_C_ associated to the gap situation are consistently larger than in the overlap situation (open circles). When going to small gate voltages, |V_GS_| < 12 V with a gap (dark grey open circles) and |V_GS_| <4 V with an overlap (black squares), the gate-induced field is too small to permit injection such that the contact voltage is approaching is maximum value |V_GS_|. As a result, the drain-source current vanishes and the device is in the off state. To switch on, the device requires a gate bias large enough so that (i) V_C_ is sufficiently large to permit injection and that (ii) an effective voltage |V_GS_|-V_C_ remains to accumulate charge carriers. This condition leads to a delayed onset of the transfer curves and, thus, to an apparent shift in the onset voltage towards more negative values^31^. This behavior is consistent with the shift in onset voltage observed for the gap operation of devices **III**-**V** (Fig. 2b).

Alongside, V_C_ gives also rise to an apparent mobility being different from the ideal mobility. We recently showed that the slope of the transfer curve ∂I_DS_/∂V_GS_, and thus the apparent mobility, contains correction contributions due to V_C_ and the change ∂V_C_/∂V_GS_ of V_C_ with gate bias. These read for a given point of operation V_DS_, V_GS_^31^.


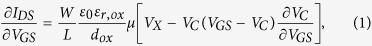


where W is the width of the device, ε_r,ox_ the dielectric constant of the dielectric layer, and ε_0_ the vacuum permittivity; V_X_ stands for V_DS_ in the linear regime (|V_DS_| < |V_GS_|) and V_GS_ in the saturation regime (|V_DS_| > |V_GS_|). The evolution of V_C_ in Fig. 4f implies that the current in gap operation must be lower than in overlap operation independent from the chosen gate bias, since both the values of V_C_ and of |∂V_C_/∂V_GS_| are larger in the gap configuration (cf. Supplementary Figure S4).

In Fig. 4f, the V_C_ values corresponding to the low mobility case are shown as well. Again, the V_C_ values found for the gap (grey open circles) markedly exceed the values in the overlap situation (filled grey squares in Fig. 4f), even though to a much lesser extent as in the high mobility case. Drastically lowered charge carrier mobilities demand a smaller space-charge limited current in the channel. To provide the matching injection current, a much smaller lowering of the injection-barrier is required. With a much lower local field strength required for injection, the device is less sensitive to a lack of source-gate overlap.

In turning back to the experimental transfer curves, we need to anticipate the consequences of spatially dependent mobilities. Due to higher fields and carrier concentrations, the field- and concentration-dependence of the mobility in disordered OSC predicts channel mobilities being larger in overlap operation than in gap operation. This does not affect the above-described situation qualitatively, yet quantitatively in a twofold way: (i) In the far linear regime (|V_DS_| ≪ |V_GS_|), i.e., where V_C_ plays a subordinate role due to intrinsically large gate-induced fields, the discrepancy between currents in overlap and gap configuration is even larger than shown in Fig. 3b,c. (ii) The shift in onset voltage is less pronounced in both configurations as the current demand of the channel is reduced due to a smaller remnant channel field (cf. Fig. 4d). Both trends are in line with the experimentally observed difference in (i) configuration-dependent onset voltages of less than 4 V and in (ii) configuration-dependent currents at V_GS_ = −12 V corresponding to a factor of 6 rather than two predicted in the simulations (Fig. 3c).

In summary, we fabricated and characterized asymmetric coplanar OTFT structures to investigate the impact of a locally impaired electric field on charge injection and transport. Owing to our self-alignment approach we established a small, tunable gap between the gate and an electrode and an overlap between gate and the counter electrode. Operating the devices with a gap to the injecting electrode yields a drain current that is at least an order of magnitude lower than the current for operating the devices with a gate-source overlap. Supported by our simulations, we attribute this behavior to the fact that charge injection across a Schottky barrier is associated to the establishment of a low-conductivity zone and voltage drop V_C_ next to the source contact. The current reduction for gap operation originates from particularly large contact voltages V_C_ as a direct consequence of a profoundly reduced gate-induced electric field near the source electrode. We demonstrate that for selected gate biases |V_GS_| > 0 the device can be in an “on-state” for overlap operation and practically in an “off-state” for gap operation. The former case can be considered weakly injection limited operation, since the contact provides an appreciable injection current with a small V_C_ penalty. In the latter case, however, the presence of a gate-source gap precludes a sufficient field-induced lowering of the Schottky barrier and, thus, injection. In this configuration, the same device at the same point of operation rather resides in a strongly-injection limited regime. This fact illustrates particularly well that the gate bias is assuming a dual role of controlling injection and accumulation in OTFTs.

## Methodology

### Fabrication and characterization of asymmetric self-aligned OTFTs

We modified the self-alignment nanoimprint lithography (SANIL) process^9^ such that the interlayer electrode overlaps lengths (i.e., on side A and B) of coplanar OTFTs can be varied systematically and changed from a gap to an overlap situation. The gate electrode is structured via nanoimprint lithography^9^. A PVCi (polyvinylcinnamate) dielectric layer is spin-coated on top. Then, the gate electrode is utilized as a mask in the photolithography step defining the positions of the source/drain electrodes. To modify the degree of overlap, the SANIL process is decisively altered in the next exposure step. The exposure angle of the sample with respect to the UV light is varied by placing the samples on 3D-printed wedges (Makerbot 2 Replicator) with different inclinations α ranging from 55° to 85° in steps of 10°. In addition, also a set of devices without wedge (nominal inclination angle 0°) was produced. The edges of the gate contact as well as the subsequently evaporated interlayer electrodes are not exactly rectangular due to scattering of UV light within the thin films. (cf. Supplementary Figure S1 for further details). In combination with using a wedge, this gives rise to variations in the source-drain separation, i.e., the channel length up to 1 μm (cf. Fig. 1c). Gold is evaporated to the prefined positions of the source and drain electrodes. Finally a pentacene layer is evaporated. The tilted samples have a smaller exposure area compared to flat lying ones, which is compensated by increasing the exposure dose from 70 mJ/cm^2^ to 560 mJ/cm^2^. For improved comparability the semiconductor is applied to all devices in one single evaporation step. In a first batch a total of 22 devices were fabricated. Since the data spread in the onset voltages of this set 1 of devices was rather large, the whole experiment was carefully repeated. A total of 13 OTFTs within set 2 with four of the five inclination angles were electrically characterized. All electrical measurements are performed in darkness under ambient conditions using an mb parameter analyzer by mb technologies.

### Determination of device geometries

For one representative device associated to each of five implemented inclinations, a cross section was prepared by focused-ion-beam (FIB) milling. Channel and overlap length are determined on a FEI NOVA 200 dual beam system in the eucentric height of 19.3 mm. To prevent charging and avoid beam damage a protective Pt/Pd layer (~80 nm, Leica EM ACE600) and a Pt/C (~1 μm) ion beam induced layer are deposited. Acceleration voltage is kept constant at 30 kV and beam currents of 500 pA for trench milling while 100 pA and 50 pA for final polishing are used with constant pixel dwell times of 500 μs. FIB processing is performed using a recently introduced interlacing patterning strategy reducing chemical damage and increasing morphological stabilities^33^,^34^, which is ideal for FIB based soft matter processing. Cross section analysis is performed *in-situ* via the electron beam using a through-the-lens-detector in secondary electron mode. Polishing and measurements have been repeated and the extracted values have been averaged.

### Modelling approach

Drift diffusion based simulations were performed on a two-dimensional transistor cross-section. The simulations comprise the self-consistent solution of the current density and continuity equation for holes and the Poisson equation. The abovementioned set of time-dependent partial differential equations are discretized on a non-uniform rectangular grid in finite difference approximation and solved using the Scharfetter Gummel method^35^. The mesh has been refined until changes in current and charge densities fell below 0.2%. The code self-consistently determines the injected hole currents by considering thermionic emission, tunneling, interface recombination, and back drift currents^17^. For each point of operation, the contact voltage is determined directly from the simulations as outlined in ref. 18. Simulations were carried out for the device geometry associated to an inclination angle α = 65° as determined by the SEM analysis (denoted below as device **IV**). The dielectric constant of the insulator, ε_r,ox_ = 2.7 was deduced from the capacitance per area C′ = 7 nF and the thickness of the insulator d_ox_ = 337 nm. The threshold voltage, defined here as the flat band condition as inspired by Meijer *et al*.^36^ was set to zero. The injection barrier height at the pentacene-gold contact was set to 0.5 eV in accord with UPS measurements^26^,^27^,^28^. This value corresponds to the offset between the hole transport level and the Fermi level in the metal and, consequently, accounts for all possible charge rearrangements between the metal surface and the organic semiconductor prior applying an external voltage, i.e., e.g. for interface dipoles or trap filling. For the sake of simplicity, we assume a constant mobility. This is mainly due to the fact that the device current depends on a subtle interplay between charge injection and transport. Thus, for more complex mobility models also a more complex injection model needs to be consistently chosen. For the particular case of field- and charge density dependent mobilities, for which analytical expressions in different scenarios are available (cf., e.g., refs 37,38), such consistent injection models are, to the best of our knowledge, not present for the relevant the injection barrier heights. We note that there are attempts in literature to mend this problem^39^; however, these do not capture relevant injection barriers for the present investigation.

## Additional Information

**How to cite this article**: Zojer, K. *et al*. Switching from weakly to strongly limited injection in self-aligned, nano-patterned organic transistors. *Sci. Rep*. **6**, 31387; doi: 10.1038/srep31387 (2016).

## Supplementary Material

Supplementary Information
