# Supplementary material for: Switching from weakly to strongly limited injection in self-aligned, nano-patterned organic transistors
Source: Sci Rep. 2016 Sep 27;6:31387. doi: 10.1038/srep31387 (PMC5037384; doi:10.1038/srep31387)
Supplement: Supplementary Information [file srep31387-s1.pdf]

Supplementary Information

Switching between weakly and strongly limited injection in self-aligned, nano-patterned organic transistors

Karin Zojer, Thomas Rothländer, Johanna Kraxner, Roland Schmied, Ursula Palfinger, Harald Plank, Werner Grogger, Anja Haase, Herbert Gold, and Barbara Stadlober**

1. Origin of the spread in gate and channel lengths

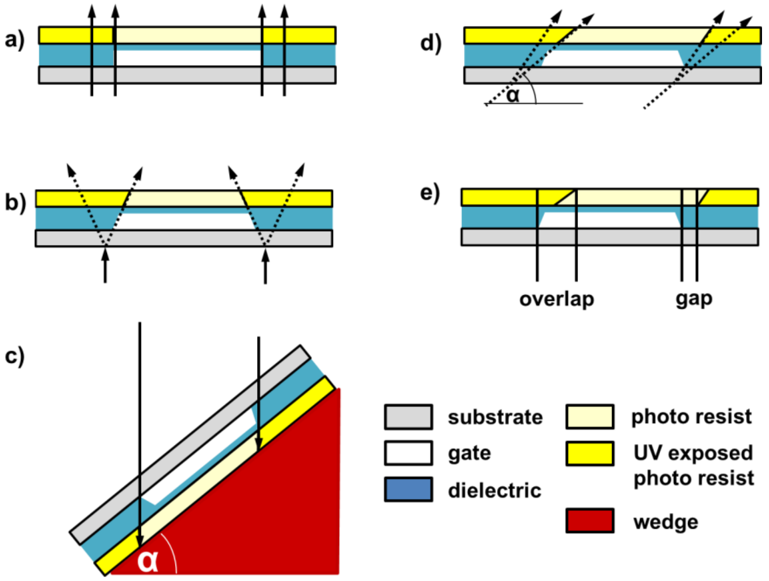

Figure S1: Schematic illustration of the tilted exposure of the photoresist defining the source and drain electrode positions. (a) Idealized exposure through the gate electrode, (b) realistic exposure through the gate electrode with UV light scattered at the layer interfaces giving rise to an overlap between gate and exposed resist, (c) tilt of exposure angle by inserting a wedge with a defined angle α, (d) origin of regions possessing a gap and an overlap between gate and exposed resist, (e) Schematic illustration of the evaluation of the gate-source and gate-drain overlap.

Figure S1 schematically illustrates the self-aligning process of a tilted sample. The edges of both the gate and, later, also of the source and drain regions are not exactly oriented perpendicular to the substrate surface, as the UV light needed to expose the photo resist is scattered at the interfaces between the layers so that there is a small spread in the exposure angle (cf. Figure S1a and b). When the sample is tilted out of the perpendicular exposure geometry to achieve an asymmetric overlap between gate to interlayer electrode (Figure S1c), the degree of the spread of exposure directions becomes dependent on the actual sample inclination α. As a result, both the resulting gate length L_G and channel length L depend on the angle α.

2. Transfer curves without correction of the onset voltage:

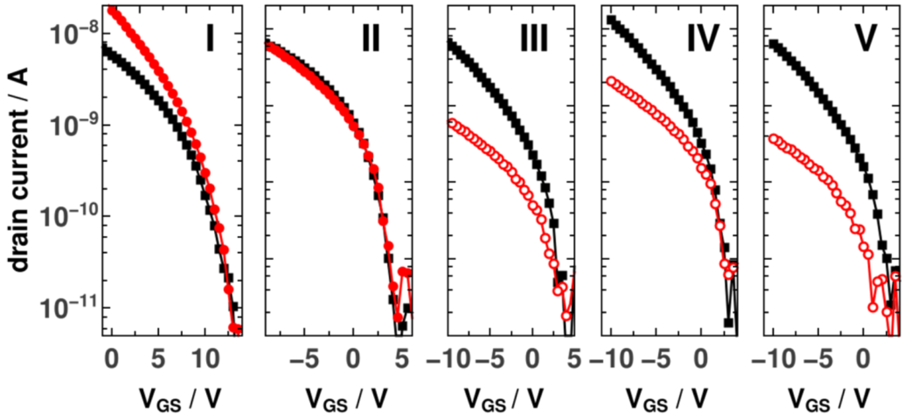

Figure S2: Transfer characteristics corresponding to Figure 2a without correction for the onset voltage. Shown is the drain current when operating the device with the source on side B (red circles), i.e., with a gap to the gate electrode (red open circles) in devices **III**-**V**, and with the source on side A (black squares). Note that devices **II** and **III** as well as **IV** and **V** have comparable channel lengths. V_{DS} = -14V for all measured curves.

3. Simulated device characteristics:

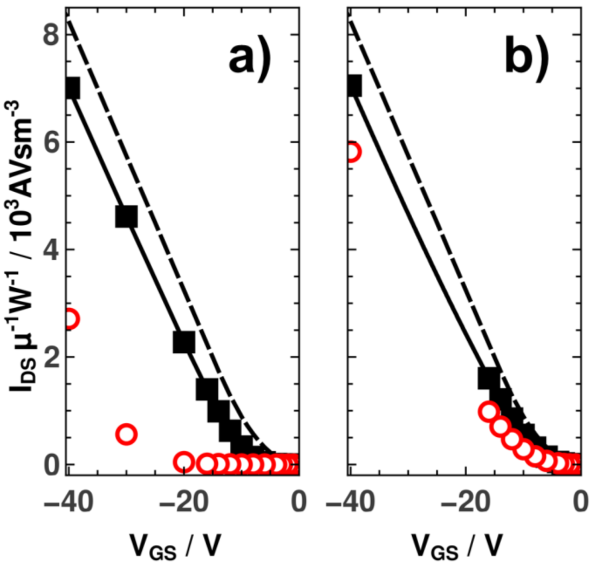

Figure S3: Scaled drain current displayed on a linear scale for the gap (red circles) and the overlap operation (black squares) as a function of the gate bias for a (a) high mobility of $\mu = 1 cm^2V^{-1}s^{-1}$ and (b) low mobility of $\mu = 10^{-3} cm^2V^{-1}s^{-1}$ and an injection barrier of 0.5 eV. For comparison, the corresponding prediction by the Gradual Channel Approximation is shown (dashed lines).

4. Contact voltage correction and apparent mobility:

To assess the impact of the contact voltage V_C on the apparent mobility, the following expression for the slope of the transfer characteristics I_{DS}-V_{GS} needs to be examined:

$$\frac{\partial I_{DS}}{\partial V_{GS}} = \frac{W}{L}\frac{\epsilon_0 \epsilon_{r,ox}}{d_{ox}}\mu \left[V_X - V_C - (V_{GS} - V_C)\frac{\partial V_C}{\partial V_{GS}}\right].$$

Here, V_X stands for the drain-source bias, V_{DS}, in the linear regime ($|V_{DS}|<|V_{GS}|$) and for gate source bias, V_{GS}, in the saturation regime ($|V_{DS}|>|V_{GS}|$). In the In presence of a contact voltage, V_C, that slope expected from the ideal characteristic, i.e., $\sim V_X$, is lowered by two V_C-containing terms entering the voltage dependence. Within the saturation regime, the two contributing factors V_C and $\partial V_C/\partial V_{GS}$ are markedly different from zero (Figure S4). The absolute values of the factors, V_C and $|\partial V_C/\partial V_{GS}|$, for gap operation (red symbols) are larger than in overlap operation (black symbols).

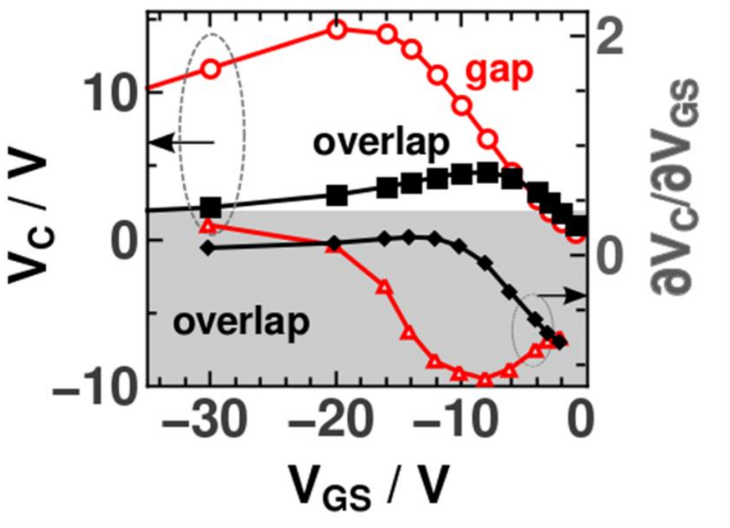

Figure S4: Contact voltage V_C and derivative $\partial V_C/\partial V_{GS}$ as a function of the gate bias for gap (red symbols) and overlap operation (black symbols) from the simulated transfer curves for μ = 1cm^2 V^{-1}s^{-1} and an injection barrier of 0.5 eV at V_{DS} = -14V (cf. Figure 3b).
